# Novel insights into the development and maintenance of the blood–brain barrier

**DOI:** 10.1007/s00441-014-1811-2

**Published:** 2014-03-04

**Authors:** Britta Engelhardt, Stefan Liebner

**Affiliations:** 1 Theodor Kocher Institute, University of Bern, Freiestrasse 1, 3012 Bern, Switzerland; 2 Institute of Neurology (Edinger Institute), University Hospital Frankfurt, Heinrich-Hoffmann-Strasse 7, 60528 Frankfurt/Main, Germany

**Keywords:** Blood-brain barrier (BBB), Tight junctions, Differentiation, BBB maintenance, BBB disruption, Wnt/β-catenin signaling, Shh signaling

## Abstract

The blood–brain barrier (BBB) is essential for maintaining homeostasis within the central nervous system (CNS) and is a prerequisite for proper neuronal function. The BBB is localized to microvascular endothelial cells that strictly control the passage of metabolites into and out of the CNS. Complex and continuous tight junctions and lack of fenestrae combined with low pinocytotic activity make the BBB endothelium a tight barrier for water soluble moleucles. In combination with its expression of specific enzymes and transport molecules, the BBB endothelium is unique and distinguishable from all other endothelial cells in the body. During embryonic development, the CNS is vascularized by angiogenic sprouting from vascular networks originating outside of the CNS in a precise spatio-temporal manner. The particular barrier characteristics of BBB endothelial cells are induced during CNS angiogenesis by cross-talk with cellular and acellular elements within the developing CNS. In this review, we summarize the currently known cellular and molecular mechanisms mediating brain angiogenesis and introduce more recently discovered CNS-specific pathways (Wnt/β−catenin, Norrin/Frizzled4 and hedgehog) and molecules (GPR124) that are crucial in BBB differentiation and maturation. Finally, based on observations that BBB dysfunction is associated with many human diseases such as multiple sclerosis, stroke and brain tumors, we discuss recent insights into the molecular mechanisms involved in maintaining barrier characteristics in the mature BBB endothelium.

## Introduction

The developing vertebrate central nervous system (CNS) derives from the ectoderm germ cell layer, a tissue that is devoid of vascular progenitor cells and that therefore lacks the capacity to generate endothelial cells. Growth of the CNS thus depends on the delivery of oxygen and nutrients from blood vessels that invade the brain from the surrounding mesoderm. Classic studies on embryonic CNS vascularization of the chick, rat and rabbit cerebral cortex by using a combination of India ink perfusion and light and transmission electron microscopy have revealed that blood vessels first form a perineural vascular plexus (PNVP) and then invade the neural tube and branch in a precise spatio-temporal manner (Bär 1983; Freeney and Watterson 1946; Strong 1964). Subsequent studies have confirmed that the development of the CNS vasculature begins via the process of vasculogenesis, whereby mesoderm-derived angioblasts invade the head region and coalescence to form the PNVP, a primitive vascular network that covers the entire surface of the neural tube at 7.5-8.5 days post-conceptionem (dpc) in mouse embryos (Hogan et al. 2004). Starting at 9.5 dpc, vascular sprouts originating from the PNVP radially invade the neuronal tube, elongage toward the ventricular zone, form a series of lateral branches at near right angles and finally anastomose with adjacent sprouts to produce a subventricular plexus of undifferentiated capillaries in the ventricular zone of the developing brain (summarized in Engelhardt 2003). Vascularization of the CNS therefore occurs by a process of new vessels being formed from pre-existing ones, i.e., by angiogenesis (Risau 1997). The rate of angiogenesis increases until the early postnatal period thus ensuring a reproducible pattern of CNS vascularization in mammals (Fantin et al. 2013; summarized in Engelhardt 2003) 

The development of BBB-defining features is not intrinsic to brain endothelial cells. Indeed, maturation of barrier characteristics in brain endothelilal cells in the vascular sprouts requires cross-talk with cellular and molecular elements from the developing CNS as shown by elegant chicken to quail (Stewart and Wiley 1981) or quail to chicken (Ikeda et al. 1996) transplantation studies, in which embryonic brain tissue from the one species was transplanted into ectopic sites of embryos of the other species. The embryonic brain tissue grafts were vascularized by sprouting angiogenesis from the non-brain host and developed BBB characteristics including the expression of BBB-specific molecules (Ikeda et al. 1996), thus providing experimental evidence that endothelial cells from invading host vessels differentiate into BBB-forming vessels under the influence of the premature neuroectoderm. Because of the unique structural relationship of astrocyte end feet ensheathing brain microvessels in the adult brain, astrocytes have long been considered as the main source of BBB-inducing signals. However, barrier induction most likely takes place well before astrocytes differentiate; hence, neuroblasts are speculated to contribute to BBB differentiation (summarized in Bauer and Bauer 2000). This barrier-inductive capacity of neuroblasts has been confirmed more recently in experiments that also implicate neuroblast-derived Wnt growth factors in this process (Lippmann et al. 2012; Weidenfeller et al. 2007). Other recent reports have, however, indicated that pericytes are necessary for the formation and regulation of the BBB (Armulik et al. 2010; Daneman et al. 2010b) and suggest that all cells of the neurovascular unit (NVU) contribute, to some extent, to the BBB phenotype of endothelial cells.

With regard to the exact time of maturation of the BBB as a physical diffusion barrier during embryonic development, data from the literature are still somewhat controversial. Early studies described the vascular sprouts invading the developing CNS as primitive sinusoids with diaphragmed fenestrae and punctate tight junctional areas; these sprouts rapidly mature during development by losing their fenestrae, developing complex tight junctional strands and forming thin-walled smaller vessels of a regular shape (summarized in Stewart 2000). In apparent contrast to these observations, vascular sprouts have been observed to be tight for serum proteins from the earliest stages of invasion into the developing CNS (summarized in Saunders et al. 2012).

## Development of the BBB

### Common angiogenic factors in CNS vascularization

**Fig. 1:**
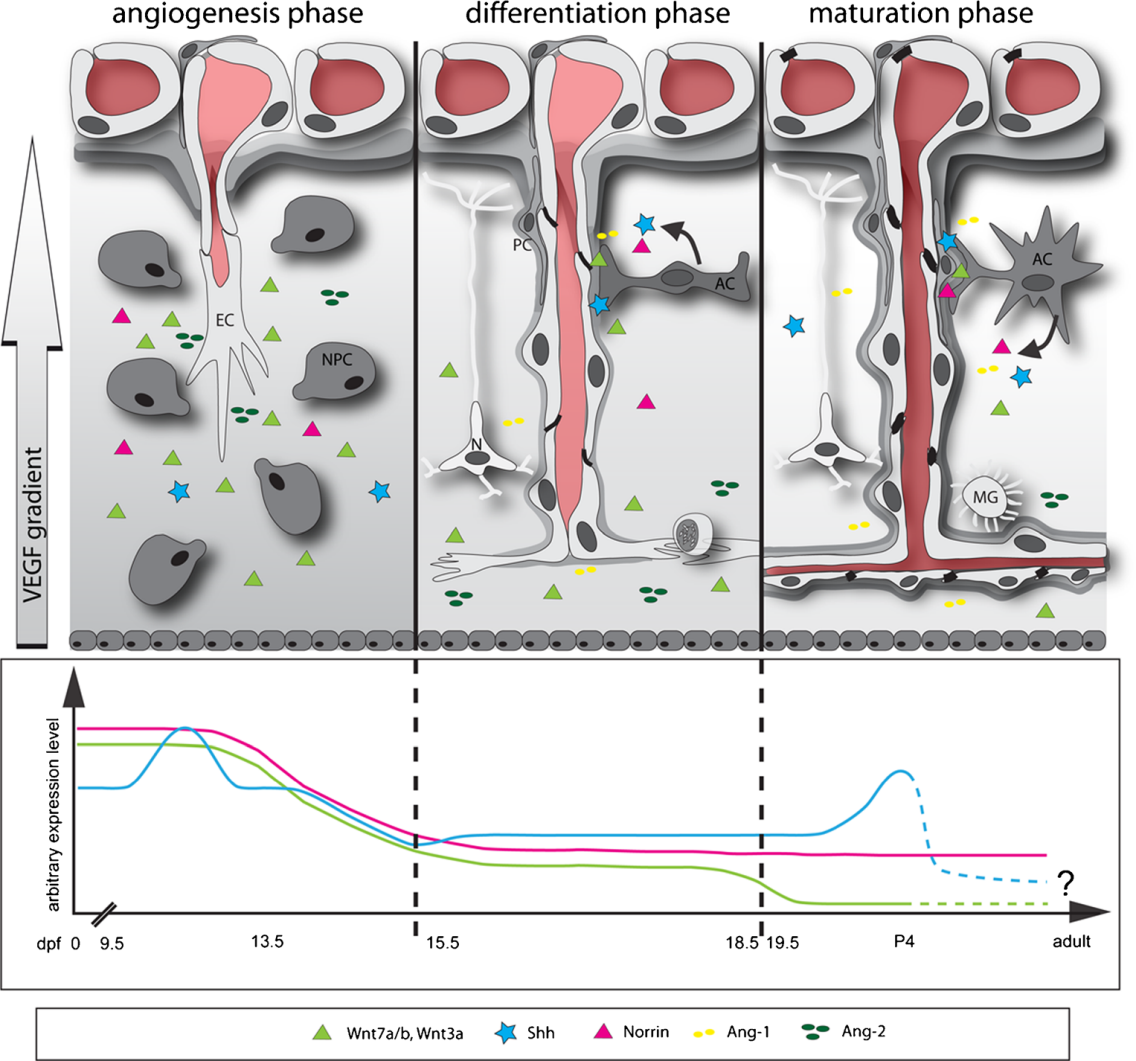
Differentiation of the blood–brain barrier (BBB). *Left* Angiogenesis phase. Vascular sprouts radially invade the embryonic neuroectoderm toward a concentration gradient of vascular endothelial cell growth factor-A (*VEGF*), which is produced by neuroectodermal cells located in the ventricular layer (*EC* endothelial cell, *NPC* neuronal precursor cell, *dpf* days post-fertilization). Growth factors such as the endothelial-cell-specific receptor tyrosine kinase Tie-2 and its ligands angiopoietin-1 (*Ang-1*) and angiopoietin-2 (*Ang-2*) are involved in angiogenic sprouting early during embryogenesis, together with the morphogens Wnt7a/7b/3a, Norrin and potentially sonic hedgehog (*Shh*). *Middle* Differentiation phase. The phenotype of cerebral endothelial cells changes such that they down-regulate their expression of the MECA-32 antigen. In this phase, the anti-angiogenic barrier-inducing signals start to overrule the pro-angiogenic signals. *Shh*, Norrin and *Ang-1* are produced by differentiating astrocytes. Tie2- and neuropilin 1 (Nrp1)-expressing myeloid cells promote anastomosis of tip cells to establish vascular circuits (*PC* pericyte, *AC* astrocyte, *N* neuronal cell). *Right* Maturation phase. Despite the cerebral endothelial cells forming the barrier proper, close contact with PCs, ACs and possibly Ns is required for the maintenance of the BBB (*MG* microglial cell, *P4* postnatal day 4). The molecular mechanisms involved in this cross-talk required for BBB maintenance in the mature central nervous system (CNS) remain largely unknown but Norrin/Frizzled4 signaling, in particular, seems to be important, at least in specific regions of the CNS (olfactory bulb, cerebellum, retina; see Fig. 2)

After its discovery in 1989, vascular endothelial cell growth factor (VEGF) and its endothelial tyrosine kinase receptors VEGFR1 (Flt-1) and VEGFR2 (Flk-1) were rapidly shown to be crucial for CNS angiognesis. VEGF is expressed and released by neural progenitors in the ventricuar neurectoderm and induces the ingrowth of capillaries from the PNVP (Raab et al. 2004). Alternative splicing from a single gene produces VEGF isoforms that differ by the absence or presence of heparin-binding domains and thus their ability to attach to the extracellular matrix. Release of these various isoforms is critical for the production of a VEGF scaffold providing spatially restricted stimulatory cues that regulate the vascular branch pattern during brain angiogenesis (Ruhrberg et al. 2002). Extracallular VEGF gradients (Fig. 1) are recognized by VEGFR2-expressing endothelial cells at the tip of the vascular sprouts forming filopodial extensions and are therefore referred to as endothelial tip cells (Gerhardt et al. 2003). Correct filopodial guidance additionally relies on the semaphorin/VEGF coreceptor neuropilin 1 (Nrp1), which specifically recognizes heparin-binding isoforms of VEGF (Gu et al. 2003). Stalk cells are endothelial cells localized in the vascular sprout behind the tip cell and proliferate in a VEGF-dependent manner, thus ensuring elongation of the vascular sprout and formation of a vascular lumen (sumarized in Geudens and Gerhardt 2011). Specification of the vascular tip versus stalk cells is mediated by the Notch signaling pathway whereby interaction of the Delta-like 4 (Dll4) ligand with Notch-1/-4 receptors inhibits tip cell and promotes stalk cell differentiation (Phng and Gerhardt 2009). Stalk cells down-regulate the expression of VEGFR2 and express higher levels of the decoy receptor VEGFR1, thus becoming less sensitive to VEGF. Interestingly, the anastomosis of neighboring tip cells to establish vascular circuits between the individual vascular sprouts has recently been shown to be promoted by macrophages expressing the receptor tyrosine kinase Tie2 and Nrp1, which invade the embryonic CNS independently of the blood vessels (Fantin et al. 2010).

Furthermore, migrating endothelial cells produce platelet-derived growth factor B (PDGF-B), which engages its receptor PDGFR-β on pericytes promoting pericyte recruitment to the immature vascular structures. Mice lacking either PDGF-B or PDGFR-β show a complete pericyte loss from brain microvessels and develop lethal microaneurisms late during embryongenesis, demonstrating that PDGF-B is actively involved in the vascularization of the brain (Lindahl et al. 1997). Precise extracellular matrix deposition of PDGF-B is essential for correct pericyte recruitment to the developing CNS vasculature, as genetic ablation of PDGF-B extracellular matrix retention sites leads to abnormal pericyte recruitment (Lindblom et al. 2003) and perturbed BBB integrity (Armulik et al. 2010; Daneman et al. 2010b), demonstrating that pericytes are involved in the differentiation and maturation of the BBB.

In addition to VEGF and Notch signaling pathways, numerous other molecular mediators of angiogenesis including the receptor tyrosine kinases Tie-1 and Tie-2, the Tie-2 ligands Angiopoietin-1 (Ang-1 and Ang−2 (Fig. 1), members of the transforming growth factor (TGF)-β superfamily and the EphB-receptor/ephrinB-ligand family together orchestrate angiogenesis in the CNS but also elsewhere in the organism (summarized in Adams and Alitalo 2007). Moreover, junction proteins such as VE-cadherin, not only contribute to vascular stability and endothelial polarity but also play a vital role in the regulation of the endothelial response to pro- and anti-angiogenic stimuli (Dejana and Vestweber 2013).

Although we have thus obtained deep insight into the mechanisms involved in brain angiogenesis, none of those mechanims solely applies to CNS angiogenesis and therefore none is a likely candidate to direct CNS endothelial cells toward their unique BBB phenotype.

### CNS-specific regulators of angiogenesis: the G-protein-coupled receptor, GPR124

The orphan G-protein-coupled receptor GPR124/TEM5 has recently been described as the first essential endothelial receptor specifically involved in CNS angiogenesis independent of VEGF (see above) and Wnt/β-catenin (see below) signaling (Cullen et al. 2011; Kuhnert et al. 2010). In the developing CNS, GPR124 is specifically expressed in the endothelium and functions cell-autonomously. Although the expression of GPR124 is not limited to specific vascular segments, GPR124 regulates endothelial cell sprouting and migration in the developing forebrain and spinal cord but not in the diencephalon, midbrain and hindbrain suggesting a corresponding embryonic spatial restriction of either GPR124 ligands or GPR124 signaling in endothelial cells and adding yet another level of complexity to CNS angiogenesis. Genetic ablation of GPR124 has demonstrated its role in regulating TGF-β signaling in a CNS-specific manner (Anderson et al. 2011). TGF-β signaling in endothelial cells has previously been shown to be involved in CNS angiogenesis, as its absence leads to aberrant vascular sprouting and hemorrhages. Pericytes and astrocytes have been identified as cellular sources for TGF-β during CNS angiogenesis (Garcia et al. 2004; Takata et al. 2007). An additional role of GPR124 beyond CNS angiogenesis in BBB differentiation is suggested by the observation that the ablation of GPR124 also reduces the expression of the BBB marker Glut-1 (Kuhnert et al. 2010; Table 1).

**Table 1:** Regulators of angiogenesis specific to the central nervous system (*CNS*) and particularly the blood-brain barrier (*TGF-β* transforming growth factor-β, *ZO-1* zonula occludens-1, *Shh* sonic hedgehog, *GPR124* G-protein-coupled receptor, *SSeCKS* *src*-suppressed C-kinase substrate, *plvap1* plasmalemma vesicle associated protein 1)

Factor	Barrier-supporting	Barrier-diminishing	Reference
β1-Integrin-mediated adhesion of endothelial cells to laminin	Expression of claudin-5		
Norrin	Up-regulation of Glut-1, claudin-5; down-regulation of plvap/meca32		Wang et al. 2012
β-Catenin	Induction of claudin-3		Liebner et al. 2008
Wnt3a/Wnt7a/b	Induction of claudin-3, Glut-1		Daneman et al. 2009; Liebner et al. 2008; Stenman et al. 2008
Shh	Immunoquiescence; regulation of claudin-5, occludin		Alvarez et al. 2011
Nogo-A		Inhibition of CNS angiogenesis	Wälchli et al. 2013
GPR124	Induction of Glut-1 TGF-β signaling in CNS endothelial cells		Anderson et al. 2011; Cullen et al. 2011; Kuhnert et al. 2010
SSeCKS	Induction of angiopoietin-1		Lee et al. 2003
Retinoic acid	Induction of pGP, ZO-1, occludin		

### CNS-specific regulators of angiogenesis and barrier genesis: morphogens regulating BBB differentiation

The morphogenic Wnt/β-catenin and hedgehog pathways are additional CNS-specific regulators of angiogenesis (Fig. 1; Alvarez et al. 2011; Liebner et al. 2011). Morphogens such as Wnt and hedgehog are considered to be mostly soluble growth factors that can act in gradients as short- or long-range factors determining cell-fate decisions during early embryogenesis and tissue formation (Table 1; Stathopoulos and Iber 2013).

Wnt proteins are secreted proteins that bind to the N-terminal cystein-rich domain (CRD) of seven-pass-transmembrane Frizzled (*Fzd*) receptors in the presence of lipoprotein-related-protein receptor 5/6 (LRP5/6). The secretion and biological activity of Wnts are highly dependent on various post-translational modifications, which, in the case of Wnts, are glycosylation on the one hand and the addition of a palmitoleic acid at conserved serine residues, on the other hand (Willert and Nusse 2012). Glycosylation appears to be crucial for the subsequent acylation that is probably conferred by the protein porcupine (*Porcn*), which is resident in the endoplasmic reticulum. At least for mouse Wnt3a, the *Porcn* is essential for a functional Wnt3a protein (Takada et al. 2006). Apparently, the post-translational modifications of Wnt growth factors also determine their solubility and thereby their biological action as diffusible cell-membrane- or extracellular-matrix-bound factors. These properties of Wnts should be kept in mind when considering their release from one cell type acting on another, as for example in the NVU. So far, the Wnt factors have been described to act in three, relatively independent pathways, known as the “canonical” β-catenin-dependent pathway and the two non-canonical pathways acting via Ca^++^/proetin kinase C (PKC)/calmodulin or c-jun N-terminal kinase (Rao and Kuhl 2010).

The β-catenin “canonical” Wnt signaling pathway (better referred to as the Wnt/β-catenin pathway) is the best described so far. Herein, the structural adherens junction protein β-catenin, which links classic cadherins via α-catenin to the actin cytoskeleton, acts as a co-transcription factor together with lymphoid enhancer factor/T-cell factor (Lef/TCF), mediating the regulation of cellular responses such as the cell cycle, apoptosis, cell differentiation and cellular communication (Klaus and Birchmeier 2008; Table 1).

In the developing CNS of vertebrates, the Wnt/β-catenin pathway has been studied in particular detail and a wide range of functions such as the determination of the midbrain-hindbrain boundary and the patterning of the telencephalon have been described. Herein, the cortical border acts as a signaling center, providing instructive Wnt cues for hippocampal development (Lee et al. 2000). Because of the many individual ligands (19 Wnt genes) and Fzd receptors (11 Fzd receptors), the differential expression of these factors has not been comprehensively studied in the developing mouse embryo in general. However, in the CNS, the expression pattern of the most-studied Wnt growth factors (Wnt-1, -3,-3a, -5a, -7a, -7b) have been described in more detail (Parr et al. 1993). Interestingly, at early time points of neural tube closure (8.0-8.5 dpc), Wnt-1, -3a and -7b are expressed in defined domains, whereas approximately 1 day later (9.0-9.5 dpc), the expression of these Wnt factors almost completely encircles the presumptive brain and spinal cord. However, individual Wnts are expressed in distinct domains, of which Wnt-1/-3/-3a are dorsally distributed, whereas Wnt-7a/-7b show a ventral expression pattern. Wnt-4 shows a unique broad dorsal distribution and, in addition, is heavily expressed in the floor plate, suggesting a specific function in dorsal and ventral differentiation. Interestingly, Daneman et al. (2009) described that, at 11.5 dpc, the expression of Wnts acquires an even more defined pattern, while maintaining the gross distribution observed at 9.5 dpc.

**Fig. 2:**
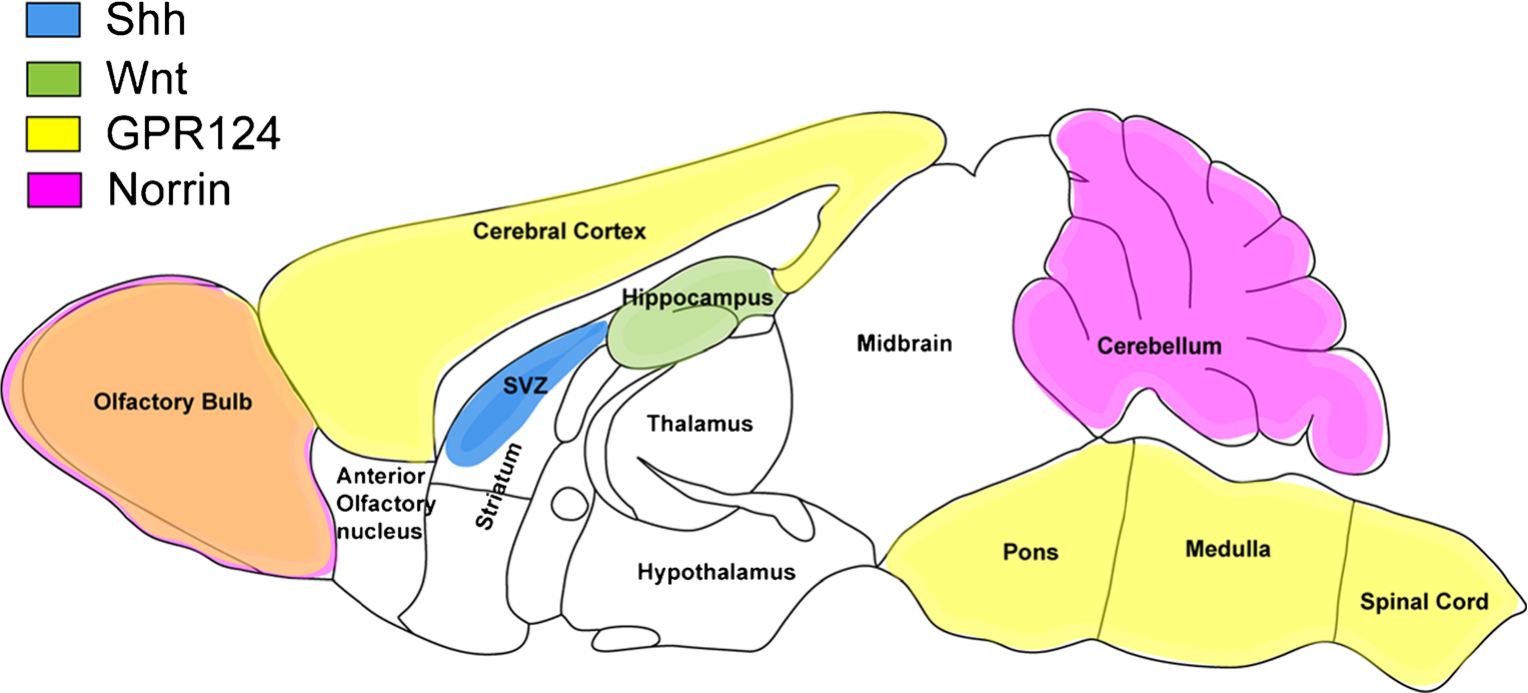
Expression of the described barrier inducing factors in the adult mouse brain. Representation of the available global expression data of barrier-inducing factors in the adult mouse brain. Note the minimal overlap but the rather distinct regional expression of the individual factors suggesting site-specific induction/maintenance cues for endothelial barrier properties in the CNS (*SVZ* subventricular zone, *Shh* sonic hedgehog, *GPR124* G-protein-coupled receptor)

In this context, the localized expression of the factors determined to date to regulate some aspect of the BBB phenotype has to be considered. As mentioned above, GPR124 is only detectable in the forebrain and spinal cord, whereas other factors such as the non-Wnt-related Fzd-4 ligand Norrin is only expressed in the retina, olfactory bulb and cerebellum. These regional differences, which are mainly detectable in the developing CNS (Fig. 2), further underline that “the BBB phenotype” probably does not exist but is instead defined by the expression of various sets of genes that make CNS microvascular endothelial cells act as an entity, unique and different from peripheral endothelial cells. Equally, the defined spatial distribution of Wnts, sonic hedgehog (Shh) and GPR124 during development further suggests that there is probably no such thing as a “one barrier-inducing pathway” but instead favors the interpretation that, depending on their final destination within the CNS, micovascular endothelial cells receive localized molecular cues, inducing “just the right” flavor of the BBB.

In vertebrates and particularly in mammals, the hedhehog (Hh) pathway is activated by the binding of one of the three identified ligands, namely sonic (*Shh*), desert (*Dhh*), or indian hedgehog (*Ihh*), to the 12-pass transmembrane receptor patched (*Ptch*). Ligand binding to Ptch releases its repression on the 7-pass transmembrane receptor smoothened (*Smo*), a member of the G-protein-coupled receptor (GPCR) family. Phosphorylation of Smo by PKA and/or G-protein–coupled receptor kinase 2 leads to the translocation of *Smo* into the membrane domain of the primary cilium, a structure present in most, if not all, vertebrate cells, rendering Smo active and leading to the conversion of *Gli* transcription factors from a repressive to an active state. In vertebrates, three *Gli* transcription factors, *Gli1*, 2 and 3, have been described, belonging to the zinc-finger family of transcription factors (Briscoe and Thérond 2013). Interestingly, the three members have distinct functions. *Gli1* is itself a direct target of Hh signaling and provides a positive feedback, with *Gli3* mainly acting as a repressor of the pathway. Current knowledge indicates that *Gli2* is the main signal-transducing transcription factor in Hh signaling.

*Shh* is probably the best-described ligand in the Hh pathway and its genetic deletion in mice leads to lethality at late embryonic or perinatal stages because of abnormalities in the eye, brain and axial patterning (Chiang et al. 1996). In humans, mutations in the *Shh* gene leads to holoprosencephaly type 3 HPE3 attributable to aberrant development of the ventral midline (Maity et al. 2005; Table 1).

The diffusible properties of the Hh ligands differ considerably from those of the Wnt factors described above, in the sense that *Shh*, for example, can form diffusible gradients over long distances. Consequently, the locally available concentration of Shh crucially influences the response of target cells, for example, in limb bud development (Lum 2004).

Interestingly, *Shh* has recently been described by Alvarez and colleagues (2011) to be released by astrocytes in the adult brain and to contribute to BBB integrity during the steady state of the BBB and, in particular, in BBB impairment during inflammatory disease. In contrast, astrocytes seem to express *Shh* especially under activated conditions, such as ischemia and experimental oxygen-glucose deprivation and to activate proliferation, migration and tube formation in brain endothelial cells via the RhoA/Rho-associated protein kinase (ROCK) pathway (He et al. 2013). Although the studies and the experimental settings (in vivo vs. in vitro) are not directly comparable, we have to understand the mechanisms of the differential effects elicited by *Shh* in CNS endothelial cells.

As a first step toward the understanding of Shh function in the adult mammalian brain in general and in BBB integrity in particular, the expression pattern of *Shh* in the adult needs to be explored in more detail at the cellular level, possibly by applying genetically inducible, fate-mapping studies for the *Shh* and/or Wnt genes (Blaess et al. 2011). So far, only little information is available regarding the expression of *Shh* in the postnatal or adult mammalian CNS. Probably the best described sites of Shh expression are the Purkinje cells of the cerebellum; these cells act on granule cells in the external germinative layer. The latter cells are sensitive to *Shh* and pathway over-activation might lead to the formation of malignant medulloblastoma (Northcott et al. 2011). However, *Shh* can also be down-regulated in the cerebellum of the adult mouse and human brain.

Interestingly, *Shh* is also involved in adult neurogenesis in the subventricular zone (SVZ), at which the factor is produced and released by specific ventral neurons and influences the generation of deep granule neurons (Ihrie et al. 2011). This finding is particularly interesting, as it has been suggested that vessels in the SVZ, which play a crucial role in adult neurogenesis by providing the so-called vascular niche, display reduced BBB characteristics (Shen et al. 2008; Tavazoie et al. 2008). Whether vessels in the SVZ respond to *Shh* released by deep granule neurons is currently a matter of speculation and needs further investigation.

In the developing CNS, however, the expression of *Shh* has been extensively studied and its expression is mainly confined to the notochord and to the floor plate at the ventral side of the neural tube, thereby defining ventral identity (Wilson and Maden 2005). In contrast, dorsal cues are provided by Wnt and bone morphogenetic proteins (BMPs), which counteract Shh signaling via the regulation of *Gli3* acting as a *Shh* transcriptional inhibitor (Ulloa and Martí 2010). In the developing CNS, Shh and Wnt growth factor expression, as far as we can judge from the available data, do not overlap on a large scale, suggesting that no systematic co-expression occurs correlating with the onset of angiogenesis and BBB differentiation in the various regions of the premature CNS.

At the cellular level, however, both *Shh* and and almost all Wnt factors are expressed by differentiated isolated astrocytes (Alvarez et al. 2011; He et al. 2013; own unpublished observations).

Notably, *Shh* has previously been reported to induce the angiogenic factor angiopoietin-1 (*Ang-1*) and to repress angiopoietin-2 (*Ang-2*), leading to increased signaling via the receptor tyrosine kinase Tie2 (Lee et al. 2007). In this regard, astrocytes have been shown to up-regulate *Ang-1* downstream of *src*-suppressed C-kinase substrate (SSeCKS), contributing to junction protein expression and vascular maturation (Lee et al. 2003; Table 1). Taking these findings together, we are tempted to speculate that Shh and SSeCKS act synergistically or in parallel in astrocytes; however, autocrine stimulation of the Hh pathway in astrocytes has not been described as yet.

The proposal that *Ang-1* is an interesting mediator of vascular stability on the one hand but also of barrier properties on the other, is further supported by a recent publication showing that *Ang-1* positively regulates β-catenin signaling in endothelial cells by phosphorylation of GSK3β via Akt, ultimately leading to the up-regulation of delta-like 4/Notch signaling, which is a master regulator of endothelial quiescence (Phng et al. 2009; Zhang et al. 2011). These findings conceptually agree with reports of Dll4 regulation by Wnt/β-catenin in developmental and pathological angiogenesis contributing to vascular maturation and quiescence (Corada et al. 2010; Reis et al. 2012).

Regarding the development of blood vessels in the CNS, Wang and colleagues (2013) more recently identified the leucine-rich alpha-2-glycoprotein 1 (*Lrg1*) as being involved in vascular growth under pathologic conditions in the retina. The authors showed that Lrg1 directly interacts with the endothelial-specific TGFβ co-receptor endoglin/CD105, thereby regulating TGFβ-induced differential activation of either smad1/5 or smad2/3 and, through this, regulating endothelial proliferation of retinal and brain endothelial cells. At this point, the contribution of *Lrg1* to brain angiogenesis and BBB differentiation is unknown but endothelial *Lrg1* deficiency might interfere with the proper formation of the brain vasculature, similar to the defects observed in endoglin/CD105 heterozygous mice, which represent a model of hereditary hemorrhagic telangiectasia type 1 (Satomi 2003).

Interestingly, as mentioned above, deficiency of GPR124 also affects angiogenesis and vascular integrity via the modulation of the TGF-β pathway in a brain-specific manner. Together, these observations suggest that CD105/endoglin and Lrg1 interact with the GPR124-TGF-β signaling axis.

A more direct and specific effect for brain angiogenesis has recently been described for the long-known axonal guidance inhibitor Nogo-A (Wälchli et al. 2013; Table 1). Briefly, the Nogo-A-pecific Delta 20 domain directly mediates endothelial growth inhibition via a Rho-A/ROCK-Myosin-II-dependent mechanism, leading to the retraction of lamellipodia and filopodia. Of interest, the effect of Nogo-A is probably more profound than just the inhibition of the lamelli-/filopodia in tip cells, as Phng and colleagues (2013) have reported that filopodia are dispensable for endothelial tip cell guidance. As *Shh* has been shown to regulate angiogenesis via the RhoA/ROCK pathway, Nogo-A might act as an antagonist of *Shh* signaling.

The observation that the developmental pathways of Wnt and *Shh* contribute to BBB induction, differentiation and eventually maintenance (see below) appears to have been a research “catalyst”, as more recently, the developmentally active retinoic acid pathway has been demonstrated to induce BBB characteristics in endothelial cells (Mizee et al. 2013; Table 1). Given the identification of the various pathways and pathway “flavors” involved in BBB induction and differentiation, the challenge for the future will be to understand their concerted action.

A first step toward this might be the recent observation that the T-box transcription factor Tbx1 is involved in the vascularization of the brain (Cioffi et al. 2013), in a strikingly similar manner to Wnt7a/b (Daneman et al. 2009; Stenman et al. 2008). Interestingly, Tbx1 is believed to be the major mutated gene in DiGeorge syndrome, a human hereditary disease leading to mental retardation attributable to vascular malformations; Tbx1 has previously been described to be downstream of Wnt/β-catenin in non-endothelial cells (Huh and Ornitz 2010).

### Barrier characteristics during development

As outlined above, the CNS acquires its vasculature solely by angiogenesis (for a review, see Liebner et al. 2011). During the angiogenesis phase, which starts in rodents at about 9.0 dpc, vessels of the PNVP cover the entire brain and start radially to invade the neuroectodermal tissue (Fig. 1). At this stage, endothelial barrier characteristics are immature with regard to the distribution of luminal and abluminal Glut-1 and the high expression of the pan-endothelial cell MECA-32 antigen (also known as plasmalemma vesicle associated protein 1, Plvap1) and with regard to the expression and localization of tight junction proteins, such as claudin-5 and claudin-3 (Liebner et al. 2011).

On the other hand, ample evidence is available showing that even the first vessels that grow into the avascular neuroectoderm are relatively tight to blood-borne proteins, such as albumin and immunoglobulin (for a review, see Saunders et al. 2012). This is in agreement with our finding that even vessels of the PNVP receive and respond to Wnt signals at 9.5 dpc, prior to entering the neuroectoderm (Liebner et al. 2008). Nevertheless, fetal brain vessels exhibit different, and in part increased, transport properties for amino acids and other metabolites, coinciding with higher demands for nutrients or a neuroprotective mechanism in the developing CNS (for reviews, see Saunders et al. 2012; Stewart 2000). Furthermore, some tight junction proteins, in particular the Wnt/β-catenin-regulated claudin-3, show increased expression and cell-cell-contact restricted localization with increasing age, specifically at postnatal stages (Liebner et al. 2008). Not ignoring the fact that the specific function of claudin-3 in endothelial cells is currently not known and the specificity for BBB still has to be determined, these findings highlight the continuous nature of BBB maturation during development.

Although blood vessels that invade the neuroectoderm during early embryogenesis do not exhibit marked leakiness to endogenous and exogenous traces of various sizes, the physiological demands on the fetal and the adult BBB are nevertheless strikingly different. This is also supported by the early postnatal lethality of claudin-5-deficient mice, which exhibit an increased leakiness for small-molecular compounds (<800 Da; Nitta et al. 2003). Apparently, this defect, i.e., leakiness, can be tolerated during embryogenesis as long as the placental barrier is in place and functional. In turn, this finding does not necessarily imply that the fetal barrier is “leaky”. However, from the view of a developmental biologist, a developing organ system is unlikely to share the same properties as the adult system, strongly indicating the existence of some degree of “immaturity”. Indeed, in the mammalian brain and in particular in rodents, angiogenic activity in the cortex well proceeds until 2–3 weeks after birth (Caley and Maxwell 1970). As angiogenic vessels do not exhibit mature properties with regard to pericyte coverage and junctional organization, BBB properties probably also require some refinement. This is further reflected by a recent unbiased screening of early postnatal versus adult brain endothelial cells, in which several hundreds of genes were found to be differentially regulated between early postnatal and adult stages, suggesting a dramatic switch in molecular and probably physiological properties (Daneman et al. 2010a). A comparision of early with late embryonic or early postnatal brain vessels would therefore be interesting, although technically challenging.

Thus, although barrier maturation in brain endothelial cells might not take place in two sequential phases as previously suggested by us (Engelhardt and Risau 1995), the recent observations discussed above support the concept of ongoing barrier genesis in brain endothelial cells during angiogenesis. Despite the long-lasting and controversial debate about the “leakiness” of the mammalian fetal BBB, this discussion turns out to be semantic rather than scientific from the current perspective, mostly because many authors still equate immaturity of the BBB with BBB leakiness during embryonic development. Thus, although “leakiness” *per se* has not been observed in recent studies, the term “immature” however appears to be appropriate, as it is objectively defined in developmental biology as a noticeable difference between developmental stages and the adult; this certainly applies to the BBB.

Once fully matured the differentiated BBB consists in a complex system formed by the highly specialized endothelial cells and their underlying endothelial basement membrane, in which a exceptionally large number of pericytes is embedded (Armulik et al. 2011). Microvessels are further enveloped by a second parenchymal basement membrane and astrocyte endfeet. Continuous cross-talk between the cellular and acellular elements of the NVU is required to control the restricted movement of molecules, ions and cells across the BBB (summarized in Engelhardt and Sorokin 2009).

## Maintenance of the mature BBB

### Cellular and molecular mechanisms maintaining BBB integrity under physiological conditions

The unique physiological features of the endothelial cells forming the BBB comprise a lack of fenestrations, low pinocytic activity and the presence of highly complex continuous tight junctions, the combination of which establishes a physical barrier. In addition, CNS microvascular endothelial cells form a metabolic barrier by the polarized expression of plasma membrane transporters either at the luminal or at the abluminal side, ensuring the transport of nutrients, ions and other metabolites into the CNS and of toxic metabolites out of the CNS (summarized in Abbott et al. 2010; Cecchelli et al. 2007).

In addition to an endothelial basement membrane harboring a large number of pericytes (Armulik et al. 2011), CNS microvessels are additionally enveloped by a second parechymal basement membrane produced by astrocytes. The astrocytes themselves tightly appose their endfeet onto the abluminal side of the CNS microvessels. This unique anatomical set-up of CNS microvessels, which is referred to as the NVU, suggests that continous cross-talk between the cellular and acellular elements of the NVU is required to control endothelial cell polarity and the restricted movement of molecules, ions and cells across the BBB (summarized in Engelhardt and Sorokin 2009). This is corroborated by the observation that, when placed in culture, CNS microvascular endothelial cells rapidly lose their unique barrier characteristics (Lyck et al. 2009), which however can be partially restored by co-culturing with pericytes or astrocytes (Coisne et al. 2005; Deli et al. 2005; Hamm et al. 2004; Wolburg et al. 1994).

In spite of the tremendous recent increase of knowledge about the molecular mechanisms that are provided by the neural enrivonment during embryonic development and that mediate BBB development and differentiation, our comprehension of the cellular sources and the molecular signals involved in maintaining the steady-state of the BBB under physiological conditions in the adult is limited to date. Expression levels of some molecules involved in CNS angiogenesis, such as the VEGF receptors, are down-regulated in adult quiescent BBB endothelium, as are their ligands in differentiated cells of the CNS parenchyma and therefore their continuous role in BBB maintenance has been questioned. Indeed, the continuous high expression levels of VEGF in the choroid plexus epithelium and of its receptors in the choroid plexus vasculature have been shown instead to maintain endothelial fenestrations in this tissue (Esser et al. 1998).

In contrast, the Wnt/β-catenin pathway might, in addition to its role in BBB development and differentiation, play a role in BBB maintenance, as suggested by recent studies performed in zebrafish and mouse models. Specifically, novel transgenic reporter zebrafish lines recently established for Wnt/β-catenin signaling convincingly demonstrate the activation of the green fluorescent protein reporter gene in adult 1-year-old animals, indicating that Wnt/β-catenin activity is at least involved in the maintenance of the CNS blood vessel network of zebrafish (Moro et al. 2012). Additional elegant studies in mice have shown that, at least in the retina and in the cerebellum, in which the non-Wnt-family Frzd-receptor agonist Norrin is expressed, adult inactivation of the gene or its cognate receptor Frzd4 leads to the loss of barrier characteristics (e.g., expression of claudin-5) and up-regulation of the permeability-associated gene plvap/meca32 (Wang et al. 2012). Together, these reports strongly suggest that the Wnt/β-catenin pathway remains operational in endothelial cells of the adult CNS, providing an essential cue for BBB maintenance. Furthermore, as certain molecular cues have been shown to display remarkable region-specific effects during CNS angiogenesis and the induction of endothelial barrier characteristics (see above) in particular, the work of Wang et al. (2012) adds this degree of complexity to BBB maintenance in the adult in which different frizzled ligands might regulate the Wnt pathway in different regions of the CNS, potentially resulting in different “flavors” of BBB characteristics. Finally, the observation that radial glia are involved in nascent vessel stabilization in the cerebral cortex by inhibiting Wnt/β-catenin signaling in CNS endothelial cells (Ma et al. 2013) adds yet another piece of knowledge to this mosaic and further underscores our superficial level of understanding of the signals and their actual signaling qualities that are responsible for maintaining BBB characteristics in the endothelium of the CNS.

At the cellular level, the polarized nature of astrocytes, as visualized by the high accumulation of the water channel protein aquaporin-4 (AQP4) in astrocyte endfeet surrounding the CNS microvessels, has been implicated in the regulation of CNS homeostasis (Liebner et al. 2011). The astrocyte endfoot is anchored via dystroglycan to the heparan sulfate proteoglycan agrin, the expression of which is up-regulated during CNS angiogenesis (Bezakova and Ruegg 2003). Nevertheless, mechanistic insights into the way that astrocyte polarity maintains BBB integrity are lacking to date.

A role for pericytes in addition to astrocytes in the maintenance of BBB characteristics has been suggested by a recent study analyzing a set of adult viable pericyte-deficient mouse mutants (Armulik et al. 2010). A comparison of these mouse mutants has shown that a decrease in pericyte coverage directly correlates with reduced barrier characteristics of CNS microvessels by increasing endothelial transcytic activity. Furthermore, this study demonstrated that, in addition to influencing BBB-specific gene expression patterns in CNS microvascular endothelial cells, pericytes also induce the polarization of astrocyte endfeet surrounding CNS blood vessels. Taken together, these observations nevertheless highlight that communication between all cellular elements of the NVU, although already implemented during development, is involved in BBB induction and maintenance. In this respect, the recent observation that ApoE and, in particular, ApoE4, which is mainly produced by astrocytes and has previously been implicated in BBB function, affects BBB function via the activation of the pro-inflammatory mediator cyclophilin A (CypA) in pericytes (Bell et al. 2010) needs to be mentioned.

Last but not least, the role of the acellular elements of the NVU in maintaining BBB characteristics in CNS microvascular endothelial cells has to be considered, especially as CNS microvessels are surrounded by two molecularly distinct basement membranes (Engelhardt and Sorokin 2009). In addition to anchoring astrocyte endfeet, pericytes and endothelial cells and thus supporting cellular polarity, the extracellular matrix within the NVU most certainly plays an important role in trapping soluble mediators such as VEGF and Wnts and thereby establishes spacial cues for BBB induction and maintenance (Kim et al. 2011).

### Cellular and molecular mechanisms impairing BBB integrity under neuropathological conditions

Loss of BBB integrity is a hallmark of a wide variety of neurological disorders including multiple sclerosis (MS), stroke, or brain tumors. Distrubance of the molecular cross-talk between the elements of the NVU in CNS pathology leads to BBB dysfunction setting the stage for edema formation, recruitment of inflammatory cells and neuronal cell death. In this context, the adhesion of cellular elements of the NVU has been recognized as a critical determinant for BBB integrity. The spatio-temporal correlation of the rapid loss of β1-integrin immunostaining and of edema formation in the ischemic core in an animal model for stroke (Tagaya et al. 2001) has suggested that β1-integrin-dependent adhesion of CNS mirovascular endothelial cells is a prerequisite for BBB integrity. Indeed, β1-integrin-mediated adhesion of brain endothelial cells to laminin in the surrounding endothelial basement membrane has been demonstrated to be critical for stabilizing claudin-5 in the tight junctions and integrity of the BBB (Osada et al. 2011). Thus, a not-as-yet described intracellular signaling pathway originating from endothelial β1-integrin engaged in binding to laminin affects the molecular architecture of the tight junctions and thus of the integrity of the BBB. Interestingly, laminins, and in particular laminin α5, have been shown to regulate *Shh* and Wnt/β-catenin signaling in a cell- and tissue-dependent manner (for a review, see Spenle et al. 2013).

Moreover, the loss of astrocyte polarity, as visualized as a loss of the polarized expression of AQP4 to astrocyte endfeet, has been observed as a hallmark in a number of CNS pathologies, including animal models of stroke (Steiner et al. 2012) and multipe sclerosis (Wolburg-Buchholz et al. 2009) and in human brain tumors (Warth et al. 2005) in which it has been demonstrated to correlate tightly with BBB dysfunction. In experimental autoimmune encephalomyelitis, an animal model of MS, leukocytes localized in the perivascular space after having penetrated the BBB secrete matrixmetalloproteases (MMP)-2 and MMP-9, leading to the degradation of the astrocyte extracellular matrix receptor dystroglycan, which is a prerequisite for leukocyte penetration across the glia limitans into the CNS parenchyma (Agrawal et al. 2006). Infiltrating leukocytes therefore destroys astrocyte foot anchorage, leading to the loss of astrocyte polarity and eventually BBB integrity. These data point to an important role of the polarized expression of astrocyte AQP4 in maintaining BBB integrity; this is further supported by the observation that BBB breakdown is a key feature of neuromyelitis optica, a neuroinflammatory disease, in which antibodies to AQP4 contribute to disease pathogenesis (Bradl et al. 2009). In apparent contrast to these observations, genetic ablation of AQP4 in mouse mutants has been observed not to correlate with BBB dysfunction (Eilert-Olsen et al. 2012; Haj-Yasein et al. 2011). These observations therefore highlight the need of inflammatory mediators in the induction of BBB distrubance, in addition to the loss of astrocyte polarity, under pathological conditions.

As pointed out above, the morphogenic *Shh* and Wnt/β-cateinin signaling pathways recently discovered to be involved in brain angiogenesis and BBB differentiation seem to play a continuous role in maintaining BBB integrity. In addition, these pathways might become re-activated under inflammatory conditions in the CNS, as they occur in MS. Hypertrophic astrocytes in active inflammatory demyelinating lesions in brain tissue from MS patients have recently been shown to display the elevated presence of Shh accompanied by the increased immunostaining of Patched-1 and nuclear localization of Gli-1 in brain endothelial cells (Alvarez et al. 2009). In addition, this study has provided direct evidence for the induction of the increased expression of components of the Shh signaling pathway in endothelial cells and astrocytes by proinflammatory cytokines.

Similar observations have been reported in ischemic brains in animal models for stroke. Specifically, *Shh* has been found to be transiently up-regulated in the focal ischemic brain in an animal model of stroke (Sims et al. 2009) and inhibition of *Shh* signaling leads to aggravated brain edema in an animal model for acute ischemic stroke (Ji et al. 2012). In this context, intracerebroventricular injections of *Shh* have been found to reduce brain edema and to preserve BBB integrity in an animal model for ischemic stroke by increasing the expression of zonula occludens-1, occludin and *Ang-1* in the ischemic penumbra (Xia et al. 2013). One is therefore tempted to speculate that neuroinflammation triggers enhanced astrocyte-endothelial cross-talk through the *Shh* pathway in an attempt to maintain and/or repair the barrier characteristics of the BBB. Furthermore, an additional feature of the *Shh* pathway observed in the context of MS is that *Shh* seems to establish the immunoquiescence of brain endothelial cells by inhibiting the expression of endothelial adhesion molecules required for immune cell trafficking across the BBB. The *Shh* pathway might therefore contribute to the maintenance of limited immune cell migration across the BBB and thus CNS immune privilege.

In brain tumors, BBB integrity is frequently lost because of tumor-induced angiogenesis and thus the formation of a nonphysiological vascular phenotype. The observation of the nuclear localization of β-catenin in blood vessels in gliobastoma has suggested an as yet unknown involvement of endothelial Wnt/β-catenin signaling in brain tumor angiogenesis (Yano et al. 2000). To address the function of endothelial Wnt signaling in glioma angiogenesis, the growth of experimental gliomas overexpressing either Wnt1 or the Wnt signaling inhibitor Dickkopf-1 were investigated in our laboratory (Reis et al. 2012). The surprising finding of these studies was that enforced activation of the Wnt/β-catenin pathway in these tumors led to reduced tumor angiogenesis by the up-regulation of Dll4 (Delta-like 4) and increased Notch signaling in endothelial cellls during tumor neoangiogenesis. As a consequence of Wnt pathway activation, the expression of tip cell genes was inhibited, whereas stalk cell genes were up-regulated, resulting in more quiescent endothelial cells. Furthermore, enforced Wnt/β-catenin signaling augmented mural cell recruitment, thereby contributing to vascular quiescence and barrier function. Although these studies suggest that reinforced Wnt/β-catenin signaling in brain tumor vessels leads to an inhibition of angiogenesis with normalized and less permeable vessels, studies in other experimental tumors highlight a proangiogenic function of the Wnt//β-catenin pathway (Min et al. 2011). Currently, the determinants of the outcome of Wnt/β-catenin signaling are unknown in the vascular compartment in general and in the BBB endothelium in particular. Whether Wnt/β-catenin will prove to be a valuable therapeutic target for anti-angiogenic and edema glioma therapy has to be determined in future studies.

## Outlook

The last few years have provided a substantial body of novel mechanistic insights into the development and differentiation of the BBB. The unique barrier characteristics established at the level of CNS microvascular endothelial cells are well established as being the result of tightly controlled cross-talk between cellular and acellular elements of the NVU. In particular, the recent discoveries of the involvement of the morphogenic wnt/β-catenin and *Shh* pathways not only in the induction, but probably also in the maintenance, of BBB characteristics in CNS endothelial cells have improved our level of understanding of the mechanisms involved in BBB maturation and maintenance. This knowledge will allow in vitro BBB models to be improved, which is urgently needed for tests of BBB permeability to chemicals or large-molecular-weight proteins, transmigration of inflammatory cells, treatments with cytokines and genetic manipulation in vitro (Paolinelli et al. 2013). In addition, this knowledge will enable CNS pathologies to be targeted by therapeutic strategies aimed at repairing the BBB. This might in principle be successful, as has been demonstrated by our recent observation that clinical benefit can be achieved in an animal model for MS by sealing BBB tight junctions through the ectopic expression of the tight-junctional protein claudin-1 in BBB endothelium (Pfeiffer et al. 2011). In this study, we found that the ectopic expression of claudin-1 at the BBB significantly reduces BBB leakiness and the disease burden during the chronic phase of the disease and hence that BBB dysfunction is a potential key event that contibutes to clinical disease in the chronic phase of neuroinflammatory disorders such as MS. The study further suggests that stabilization of BBB function will be beneficial in treating neuroinflammatory diseases (Pfeiffer et al. 2011).

Improved in vitro BBB models and inducible transgenic mouse models will allow the delineation of the molecular mechanisms involved in BBB differentiation and maintenance and the discovery of mechanisms dysregulated in CNS pathology leading to BBB dysfunction. The challenge ahead is to unravel the spatio-temporal involvement of distinct molecular cues and their cellular sources, e.g., pericytes, astroglial cells and neuroblasts, in inducing and maintaining BBB characteristis in CNS microvascular endothelial cells in vivo.
